# Prenatal Choline Supplementation during High-Fat Feeding Improves Long-Term Blood Glucose Control in Male Mouse Offspring

**DOI:** 10.3390/nu12010144

**Published:** 2020-01-04

**Authors:** Hunter W. Korsmo, Kaydine Edwards, Bhoomi Dave, Chauntelle Jack-Roberts, Huanling Yu, Anjana Saxena, Marie Salvador, Moshe Dembitzer, Jaskomal Phagoora, Xinyin Jiang

**Affiliations:** 1 PhD Program in Biochemistry, The Graduate Center CUNY (City University of New York), New York, NY 10016, USA; hkorsmo@gradcenter.cuny.edu (H.W.K.); asaxena@brooklyn.cuny.edu (A.S.); 2 Department of Health and Nutrition Sciences, Brooklyn College of the City University of New York, Brooklyn, NY 11210, USA; kaydineedwards@outlook.com (K.E.); bhoomiadave@gmail.com (B.D.); chauntelle.r@gmail.com (C.J.-R.); slvdr.marie@gmail.com (M.S.); 3 Department of Public Health, Capital Medical University, Beijing 100069, China; yuhlzjl@ccmu.edu.cn; 4 Department of Biology, Brooklyn College of the City University of New York, Brooklyn, NY 11210, USA; moshe.dembitzer@macaulay.cuny.edu; 5 Department of Chemistry, Brooklyn College of the City University of New York, Brooklyn, NY 11210, USA; jaskomalphagoora@gmail.com

**Keywords:** choline, maternal obesity, blood glucose, adipose tissue, insulin signaling

## Abstract

Maternal obesity increases the risk of metabolic dysregulation in rodent offspring, especially when offspring are exposed to a high-fat (HF), obesogenic diet later in life. We previously demonstrated that maternal choline supplementation (MCS) in HF-fed mouse dams during gestation prevents fetal overgrowth and excess adiposity. In this study, we examined the long-term metabolic influence of MCS. C57BL/6J mice were fed a HF diet with or without choline supplementation prior to and during gestation. After weaning, their pups were exposed to either a HF or control diet for 6 weeks before measurements. Prenatal and post-weaning dietary treatments led to sexually dimorphic responses. In male offspring, while post-weaning HF led to impaired fasting glucose and worse glucose tolerance (*p* < 0.05), MCS in HF dams (HFCS) attenuated these changes. HFCS (versus maternal normal fat control) appeared to improve metabolic functioning of visceral adipose tissue during post-weaning HF feeding, preventing the elevation in leptin and increasing (*p* < 0.05) mRNA expression of insulin receptor substrate 1 (*Irs1*) that promotes peripheral insulin signaling in male offspring. In contrast, MCS had minimal effects on metabolic outcomes of female offspring. In conclusion, MCS during HF feeding in mice improves long-term blood glucose homeostasis in male offspring when they are faced with a postnatal obesogenic environment.

## 1. Introduction

In the US, over 60% of women in their reproductive age are overweight or obese [1]. Maternal obesity not only increases the risk of metabolic abnormalities during pregnancy, such as gestational diabetes mellitus (GDM), resulting in fetal overgrowth or macrosomia at birth, but also exerts long-lasting impacts on the offspring, increasing their risks for obesity, diabetes, and other cardio-metabolic diseases in adulthood [1,2].

Maternal dietary intakes provide an opportunity to overcome the negative influence of maternal obesity and a postnatal obesogenic environment on offspring health. Choline is a versatile semi-essential nutrient contained in eggs, meat, beans, and other foods. It participates in lipid metabolism, membrane structure maintenance, methyl group donation, and cellular signaling [3]. Research has shown that choline supplementation during pregnancy alters fetal epigenetic programming, improves offspring cognitive development, and modifies placental vasculature and macronutrient transport in rodents or humans [3]. We previously reported that choline supplementation during pregnancy prevented fetal overgrowth and alleviated excess adiposity in high-fat (HF) fed mouse dams that were obese and glucose intolerant [4,5]. We proposed a mechanism where choline supplementation during maternal obesity and GDM reduced macronutrient anabolism and glucose/fat transport to the fetus by mitigating the mTOR (mechanistic target of rapamycin) signaling pathway in the placenta, leading to reduced fetal overgrowth [4]. Maternal choline supplementation (MCS) also appeared to downregulate the expression of lipogenic genes in the liver of fetuses from HF dams, serving as a plausible mechanism to reduce liver triglyceride accumulation in these fetuses [5]. Specifically, choline supplemented fetuses had lower hepatic mRNA expression of acetyl-CoA carboxylase 1 (*Acc1*), fatty acid desaturase 1 (*Fads1*), and ELOVL fatty acid elongase 5 (*Elovl5*), as well as sterol regulatory element-binding transcription factor 1 (*Srebf1*), which activates the transcription of lipogenic genes, when compared to unsupplemented HF fetuses [5]. It is unclear whether these alterations in fetal liver gene expression by maternal choline supplementation can be maintained into adulthood and influence long-term metabolic health of the offspring.

Maternal HF feeding has been demonstrated to result in long-term metabolic dysregulation in the rodent offspring, such as steatosis, even when offspring are fed a normal low-fat diet after weaning [6]. Exposing offspring to a postnatal HF diet in addition to maternal HF feeding further exacerbates metabolic abnormalities, resulting in obesity [7,8], increased food intake [7], steatohepatitis [9], inflammation [8,10], hyperglycemia [11], and impaired insulin signaling in central and peripheral tissues [7]. Underlying the phenotypic alterations in HF offspring are gene expression changes, such as the upregulation of phosphoenolpyruvate carboxykinase (PEPCK) that mediates gluconeogenesis [12] and stearoyl-Coenzyme A desaturase 1 (SCD1) [13] that mediates lipogenesis in the liver, and downregulation of the peroxisome proliferator-activated receptor gamma (PPAR-γ) [7], a critical regulator of peripheral insulin resistance that promotes triglyceride storage and reduces free fatty acid (FFA) release in the white adipose tissue. MCS may influence offspring metabolic gene expression, thereby modulating lipid homeostasis and insulin signaling in the long term.

Studies also demonstrate that over a quarter of gene expression changes in offspring exposed to maternal HF feeding are associated with DNA methylation alterations [14], suggesting that epigenetic modifications early in life are likely contributors to later metabolic abnormalities. Choline, as a methyl donor, has been demonstrated to alter global and/or site-specific DNA methylation in both human and mouse placentas [15,16] and offspring tissues [16,17,18,19]. Hence, MCS may also interact with maternal HF feeding to influence offspring metabolism via an epigenetic mechanism.

In this study, we aimed to examine the long-term influence of MCS on modifying adiposity and blood glucose control of offspring that were also exposed to maternal obesity and GDM prenatally. Given that the postnatal environment may attenuate or exacerbate cardio-metabolic phenotypes, we explored the differential effects of MCS on offspring outcomes when they were exposed to either a normal post-weaning feeding environment or HF-feeding. We analyzed the data in male and female offspring separately, to address the potential sexually dimorphic responses of offspring metabolism to pre- and post-natal dietary treatments.

## 2. Materials and Methods

### 2.1. Animals and Diets

**Figure 1:**
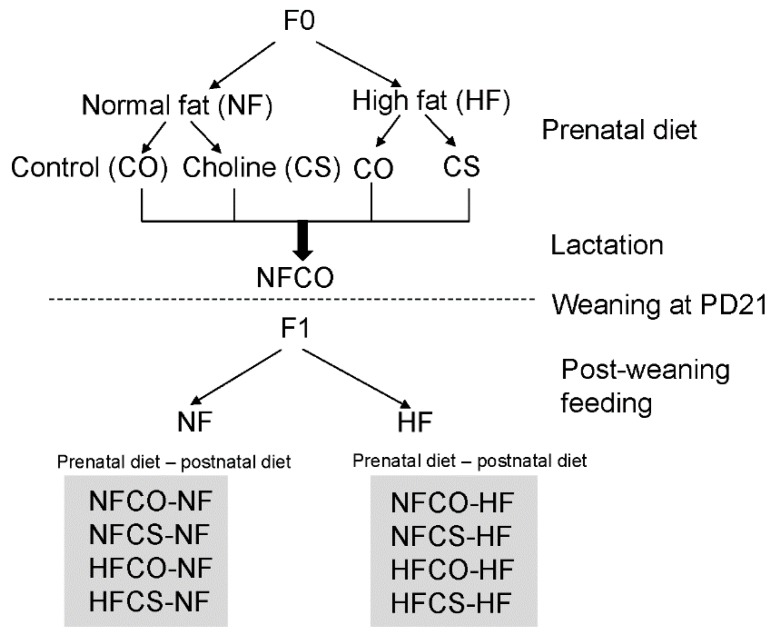
Study design. F0: mouse dams; F1: mouse offspring; NF: normal fat (10%kcal); HF: high fat (60% kcal); CO: untreated control without choline; CS: choline supplemented; PD: postnatal day.

The study protocol was approved by the Institutional Animal Care and Use Committee (IACUC) at Brooklyn College. C57BL/6J mice were obtained from the Jackson Laboratory originally and were bred in the Brooklyn College animal facility. The mice were housed at 22 °C, humidity 40–60%, and 12-h light/dark cycle with regular bedding and enrichment. The mice were fed a regular lab diet (Laboratory Rodent Diet 5012, LabDiet, St. Louis, MO, USA) ad libitum until 8 weeks of age. Female and male mice (F0) were caged together in a 2:1 ratio for mating. The male mice were removed to separate cages after 7 days. After the pups were born, female mice nursed the pups until weaning at postnatal day (PD) 21. Good breeders that successfully raised the pups were selected for further experiments. Two weeks after weaning of pups, F0 female mice were divided into 4 groups: the normal-fat control (NFCO) group received a normal-fat (NF) diet (D12450J, Research Diets, New Brunswick, NJ, USA) containing 10% kcal from fat and purified drinking water; the NF choline supplemented (NFCS) group received the NF diet and purified drinking water supplemented with 25 mM of choline chloride; the high-fat control (HFCO) group received a high-fat (HF) diet (D12492, Research Diets) containing 60% kcal from fat and purified drinking water; and the HF choline supplemented (HFCS) group received the HF diet and purified drinking water supplemented with 25 mM of choline chloride (Figure 1). Male mice for mating received the NFCO diet. The composition of experimental diets was previously described by others [20,21] and we reported the total choline contents in these diets (11.7 mmol/kg in the HF diet and 7.6 mmol/kg in the NF diet) previously [4]. The 60% kcal HF diet was chosen based on our prior studies demonstrating stable phenotypes of obesity and glucose intolerance in dams [4]. Others have also reported that this HF diet can consistently lead to obesity even for female C57BL/6J mice that are less susceptible to diet-induced obesity than male mice [22]. The 60% kcal fat is higher than the fat intake in a typical human diet. However, this fat intake is achievable in people following a HF diet regimen, such as the modified Atkins diet and the ketogenic diet (60–90% kcal fat) [23]. The HF and NF diets have equivalent levels of protein and micronutrients per kcal and thus the HF diet is not protein or micronutrient-restricted. The n-3 to n-6 fatty acid content ratio was also similar between the two diets. We recorded food and water intake of animals each week. Based on the intake and choline concentration in water and food, the level of choline supplementation yielded 4.5 times total choline intake in the choline supplemented versus control groups, which has been reported in our previous publication [4]. This supplementation level was demonstrated to improve offspring cognitive development and placental functioning in several studies [4,24,25,26]. In humans, four times higher choline intake than the Adequate Intake (AI, 450 mg/day for pregnant women) level is achievable by a choline supplement and is within the range of recommended intakes below the upper tolerable intake level of 3500 mg/day [27].

After 4 weeks of feeding with experimental diets, female and male mice (F0) were caged together in a 2:1 ratio for timed-mating. If a vaginal plug was detected in the morning, the female mouse was transferred to a separate cage and time was recorded as embryonic day 0.5. Female mice continued to receive their assigned diets during gestation. As HF dams were less successful in keeping their pups (F1) during lactation, to increase the survival rate of HF pups, all female mice were provided with the NFCO diet during lactation until weaning of pups at postnatal day 21. This design also allowed us to pinpoint whether choline exposure during the prenatal period was enough to generate differences in metabolic response. After weaning, two male and two female offspring were randomly chosen from each litter and fed either a NF or HF diet without choline supplementation for 6 weeks (Figure 1). Weight gain and food intake of F1 mice were monitored weekly during post-weaning feeding. Blood glucose was measured with a glucometer at week 0 and 6 of post-weaning feeding. The post-weaning NF (PWNF) groups exposed to varied maternal diets contained at least six litters or twelve F1 animals of each sex per group, while the post-weaning HF (PWHF) groups contained at least eight litters or sixteen F1 animals of each sex per group.

### 2.2. Intraperitoneal Glucose Tolerance Test (IGTT)

The IGTT was conducted on the F1 mice after 6 weeks of post-weaning feeding, as previously described [4]. Blood glucose levels were checked at baseline 0, 15, 30, 60, 90, and 120 min after glucose injection.

### 2.3. Sample Collection

After 6 weeks of post-weaning feeding, F1 mice were euthanized by carbon dioxide inhalation after 6-h fasting. Blood samples were retrieved by cardiac puncture immediately after euthanasia and collected into a serum separator tube (BD, Franklin Lakes, NJ, USA) and centrifuged at 12,000× *g* for 10 min to obtain serum. The liver, inguinal fat, gonadal fat, and mesenteric fat were dissected, rinsed in phosphate buffered saline, and dried on absorbent paper. The samples were then weighed on an analytical balance. Thereafter, they were either flash frozen in liquid nitrogen and stored at −80 °C or immersed in RNAlater^®^ (Thermo Scientific, Grand Island, NY, USA) overnight before being stored at −80 °C until analysis.

### 2.4. Biomarker Measurements

Serum biomarkers of F1 mice, including insulin, adiponectin, and leptin were measured in one male and one female from each litter with enzyme-linked immunosorbent assay (ELISA) kits (insulin and leptin: Alpco, Salem, NH, USA; adiponectin: RayBiotech Life, Peachtree Corners, GA, USA). Serum and liver triglyceride concentrations were measured with the Triglyceride Colorimetric Assay Kit (Cayman, Ann Arbor, MI, USA) and serum free fatty acids were measured with the HR Series NEFA-HR [2] colorimetric reagents (Wako Diagnostics, Richmond, VA, USA) according to the manufacturers’ instructions.

### 2.5. RNA Extraction and Quantitative Real-Time PCR

RNA was extracted from one female and one male liver or gonadal fat sample from each litter using the TRIzol^®^ reagent (Thermo Scientific). Reverse transcription was conducted using the High-Capacity cDNA Reverse Transcription kit (Thermo Scientific) following the manufacturer’s instructions. Gene transcript abundance was analyzed by quantitative real-time PCR with SYBR green detection using the CFX384 Touch™ Real-Time PCR Detection System (Bio-Rad, Hercules, CA, USA) as previously described [4]. Data were expressed as the fold difference of the gene of interest relative to the housekeeping gene, beta-actin (*Actb*) using the 2^−ΔΔCt^ method [28]. All primers were designed using GeneRunner Version 3.01 [29] (Table S1). Expression of the following genes was analyzed: *Acc1*, *Acc2*, peroxisomal acyl-coenzyme A oxidase 1 (*Acox1*), carnitine palmitoyltransferase 1a (*Cpt1a*), carbohydrate-response element-binding protein (*Chrebp*), fatty acid transporter 1 (*Fatp1*), *Elovl5*, glucose transporter 2 (*Glut2*), *Glut4*, insulin receptor substrate 1 (*Irs1*), monocyte chemoattractant protein-1 (*Mcp1*), microsomal triglyceride transfer protein (*Mttp*), PEPCK (*Pck1*), PPAR-α (*Ppara*), PPAR-γ (*Pparg*), resistin (*Retn*), retinol binding protein 4 (*Rbp4*), scavenger receptor class B member 1(*Scarb1*), *Scd1*, *Srebp1c*, and tumor necrosis factor alpha (*Tnfa*).

### 2.6. Statistical Analyses

The dataset was stratified by sex of the offspring (F1) because previous studies suggest that male and female offspring had different metabolic responses to prenatal exposures [8,30]. General linear models (GLMs) followed by post-hoc Tukey’s HSD tests were constructed to assess the differences in the dependent variables including offspring weight at weaning and end of study, fat pad and liver weight, and blood glucose concentrations with maternal dietary intake group and post-weaning dietary intake group and their two-way interaction as independent variables. If the maternal diet and post-weaning diet interaction had a *p* value less than 0.1, we stratified the data by post-weaning diet to analyze the effect of maternal diet on dependent variables under the PWHF and PWNF feeding conditions separately. Post-weaning food intake over time, offspring weight gain over time, and glucose tolerance were analyzed with repeated measures GLM using the same independent variables and method as above. Other dependent variables, including serum biomarkers, liver and gonadal fat metabolites, and gene expression, were compared with the one-way analysis of variance (ANOVA) test followed with host-hoc pair-wise comparisons among the four PWHF groups, i.e., NFCO-HF (prenatal normal fat control—postnatal high fat), NFCS-HF (prenatal normal fat choline supplemented—postnatal high fat), HFCO-HF (prenatal high fat control—postnatal high fat), HFCS-HF (prenatal high fat choline supplemented—postnatal high fat), and the absolute control group NFCO-NF (received the NF diet without choline during both prenatal and postnatal feeding). Dependent variables with residuals that deviate from the normal distribution were logarithmically transformed before analysis. A *p* value < 0.05 was considered as significant. Values are presented as means ± standard error of mean (SEM).

## 3. Results

### 3.1. MCS Decreases Food Intake during Postnatal HF Feeding in Male Offspring

**Figure 2:**
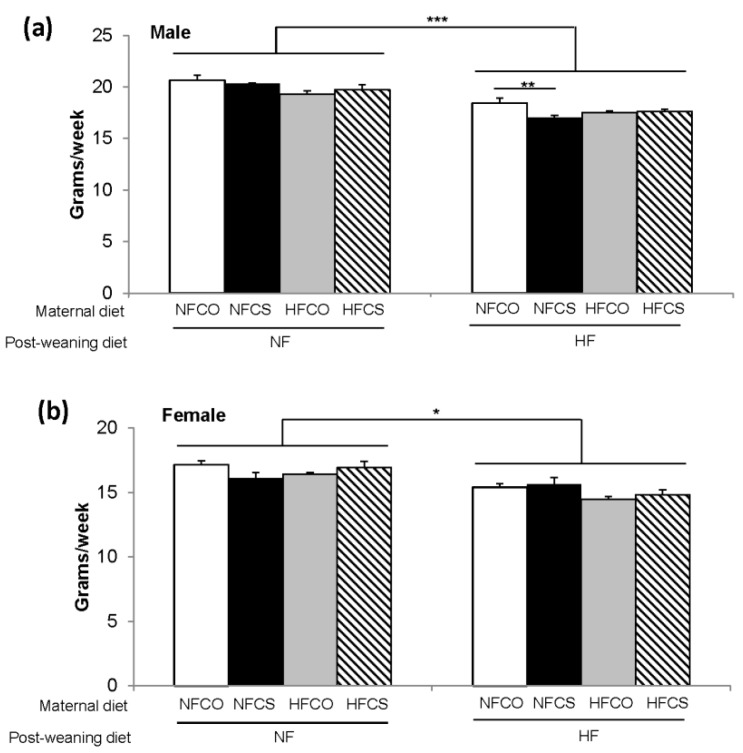
Average food intake during the 6-week post-weaning feeding. (**a**) Represents males and (**b**) represents females. Open bar: NFCO, closed bar: NFCS, grey bar: HFCO, hatched bar: HFCS. n = 12 for the postnatal NF groups and *n* = 16–20 for the postnatal HF groups for each sex. Data were analyzed using the general linear model. Values represent means ± SEM. *, *p* < 0.05; **, *p* < 0.01; ***, *p* < 0.001; NF: normal fat; HF: high fat; CO: untreated control without choline; CS: choline supplemented.

Maternal and post-weaning diets interacted to affect food and calorie intakes during the 6-week post-weaning feeding in both male and female offspring (*p* < 0.05). For both male and female offspring, PWHF (post-weaning high fat) feeding decreased food intake compared to PWNF (post-weaning normal fat) (*p* < 0.001). However, because the HF diet has a higher energy density than the NF diet (5.21 versus 3.82 kcal/g), the PWHF animals had significantly higher caloric consumption than the PWNF animals (91.9 ± 0.7 versus 77.2 ± 0.8 kcal/week in males and 78.6 ± 0.8 versus 64.1 ± 0.9 kcal/week in females, *p* < 0.001). After stratifying the data by post-weaning diet, the NFCS-HF (prenatal normal fat choline supplemented—postnatal high fat) male offspring had lower (*p* = 0.003) food intake than the NFCO-HF (prenatal normal fat no choline control—postnatal high fat) male offspring (Figure 2a). The calorie intakes were lower in the NFCS-HF than the NFCO-HF group accordingly. Maternal diet did not affect post-weaning food or calorie intake in female offspring (Figure 2b).

### 3.2. Maternal HFCS Alleviates Faltered Offspring Growth during Lactation

**Figure 3:**
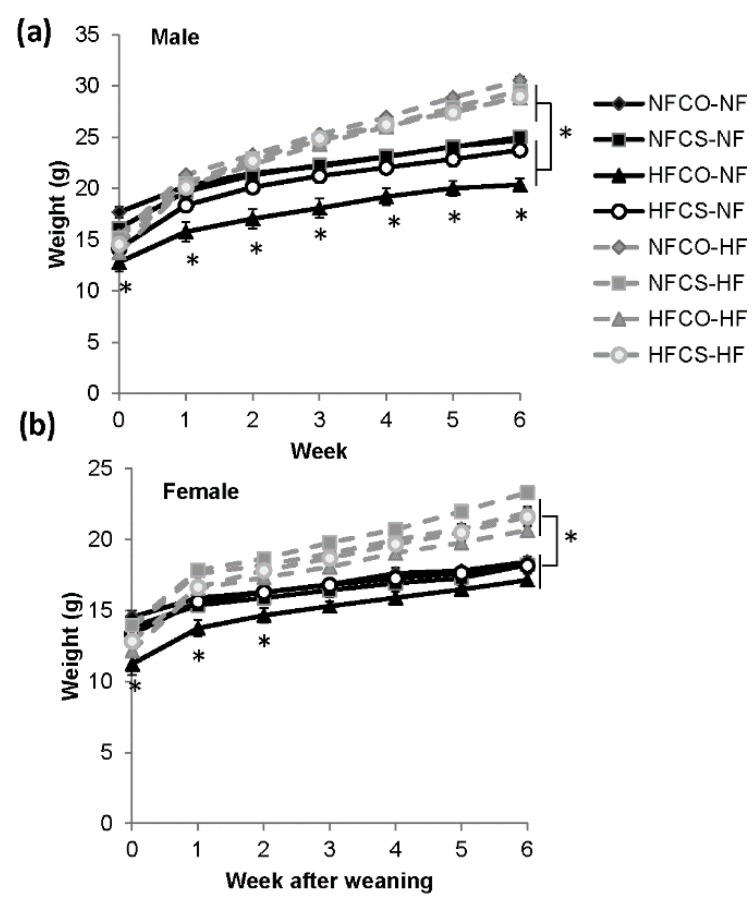
Mouse offspring weight during the 6-week post-weaning feeding. (**a**) Represents males and (**b**) represents females. Grey and dashed lines: post-weaning NF feeding groups, black and solid lines: post-weaning HF feeding groups. *n* = 12 for the postnatal NF groups and *n* = 16–20 for the postnatal HF groups for each sex. Data were analyzed using the general linear model. Values represent means ± SEM. *, *p* < 0.05; NF: normal fat; HF: high fat; CO: untreated control without choline; CS: choline supplemented.

We then compared the weight gain of offspring during the 6-week post-weaning feeding. At weaning of the offspring (week 0) and before initiation of post-weaning feeding, both the female and male offspring in the prenatal HFCO (high fat control) group had lower body weight than the prenatal NF groups (*p* < 0.05) (Figure 3), suggesting that prenatal HF feeding paradoxically inhibited the growth of pups during the suckling period, whereas MCS (maternal choline supplementation) in the HFCS (high fat choline supplemented) group appeared to alleviate the growth inhibition of the offspring (*p* > 0.05 compared to the NF groups).

During the 6-week post-weaning feeding, weight gain of male offspring demonstrated an interaction between maternal and post-weaning feeding (*p* < 0.01). In these male offspring, PWHF led to higher weight gain than PWNF (*p* < 0.01). After stratifying the data by post-weaning diet, the prenatal HFCO offspring still had lower body weight than the other groups when fed the PWNF diet (HFCO-NF vs. other PWNF groups, *p* < 0.05) (Figure 3a). However, in the PWHF environment, male offspring from all prenatal dietary treatment groups gained similar weight, suggesting that the constraint of maternal HF feeding on postnatal growth was eliminated by PWHF.

In female offspring, PWHF led to higher body weight (*p* < 0.01) than PWNF (Figure 3b). The lower body weight of the HFCO offspring at weaning due to maternal HF feeding was eliminated after post-weaning feeding in both the PWNF and PWHF environments.

### 3.3. Maternal HFCS Improves Blood Glucose of Male Offspring Exposed to an Obesogenic Diet

**Figure 4:**
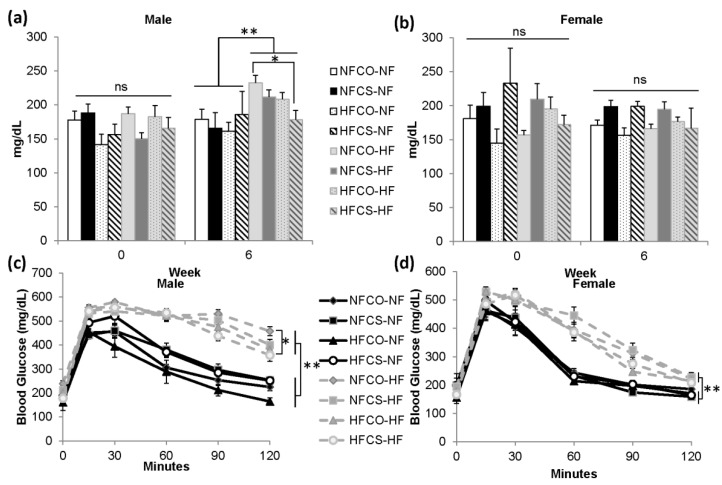
Blood glucose control of mouse offspring during the 6-week post-weaning feeding. (**a**,**b**) Fasting blood glucose at week 0 and week 6 of post-weaning feeding in male and female offspring. (**c**,**d**) intraperitoneal glucose tolerance at week 6 of post-weaning feeding in male and female offspring. *n* = 12 for the postnatal NF groups and *n* = 16–20 for the postnatal HF groups for each sex. Data were analyzed using the general linear model. Values represent means ± SEM. *, *p* < 0.05; **, *p* < 0.01; NF: normal fat; HF: high fat; CO: untreated control without choline; CS: choline supplemented.

We measured fasting blood glucose levels at week 0 and 6 of post-weaning feeding. At week 0, before initiation of post-weaning feeding, there were no differences in blood glucose levels among prenatal dietary treatment groups in either sex (Figure 4).

At week 6 of post-weaning feeding, we observed a trend where maternal and post-weaning diets interacted to influence blood glucose measurements (*p* < 0.1) in male offspring. PWHF overall led to higher blood glucose than PWNF (*p* < 0.01) in these male offspring (Figure 4a). However, the HFCS-HF animals had significantly lower glucose levels than the NFCO-HF animals (*p* = 0.015) and normalized their glucose levels to those that were comparable to the NFCO-NF group (*p* = 0.9), suggesting that prenatal HFCS alleviated the elevation in blood glucose among male offspring that were exposed to post-weaning HF feeding (Figure 4a). There were no differences in blood glucose among female offspring due to either maternal or post-weaning diet at this time point (Figure 4b).

We also measured glucose tolerance at 6 weeks of post-weaning feeding. PWHF feeding worsened blood glucose tolerance in both male and female offspring (*p* < 0.01). In male offspring, prenatal choline supplementation in the HFCS-HF group mitigated the rise in blood glucose intolerance due to PWHF, when comparing with the NFCO-HF group (*p* = 0.04) (Figure 4c). Maternal diet, on the other hand, did not affect glucose tolerance among female offspring (Figure 4d).

### 3.4. Offspring Liver Triglycerides and Gene Expression Are Not Altered by MCS

Since we observed limited differences in weight or glucose tolerance in the PWNF offspring, in the following sections, we focus our analyses on the PWHF animals exposed to different maternal diets. We also include the NFCO-NF group as an absolute control in which the animals were exposed to a control NF environment without choline supplementation both prenatally and postnatally.

Since the liver is that a critical organ regulates blood glucose and lipid metabolism, and the main location of choline metabolism, we then measured triglyceride content and metabolic gene expression in this organ. In male offspring, liver triglyceride content did not differ among the groups, while female offspring in both the NFCO-NF and HFCO-HF groups revealed lower liver triglyceride concentrations than the other groups (*p* = 0.023) (Table 1).

Liver gene expression, including those related to fatty acid synthesis (*Acc1*, *Acc2*, *Scd1*, *Fads1*, *Elovl5*), fat acid transport (*Fatp1*), fatty acid β-oxidation (*Ppara*, *Acox1*, *Cpt1a*), lipoprotein metabolism (*Scarb1*, *Mttp*), gluconeogenesis (*Pck1*) and glucose uptake (*Glut2*), or transcriptional control of glucose and fat metabolism (Shrebp1c, Chrebp1), did not differ among the PWHF groups (Figure S1). However, PWHF led to lower expression of lipogenic genes (*Acc2*, *Scd1*) in both males and females when comparing to the NFCO-NF group (*p* < 0.05). In male mice, PWHF also reduced (*p* < 0.05) the expression of the gluconeogenic gene *Pck1* and the expression of *Mttp* and *Scarb1*, which mediate very-low-density lipoprotein (VLDL) assembly and high-density lipoprotein (HDL) uptake, respectively. In female mice, PWHF also reduced expression of the lipogenic gene *Acc1* (*p* = 0.014).

### 3.5. MCS Prevents the Elevation in Serum Leptin Due to PWHF in Male Offspring

We then investigated whether alterations in extrahepatic tissues in response to dietary treatments may explain the differences in offspring blood glucose control. We measured serum concentrations of triglycerides and FFAs, as well as a few hormones and cytokines, i.e., insulin and the adipokines leptin and adiponectin, which are regulators in the adipo-insular axis that affect peripheral insulin sensitivity.

In male offspring, serum insulin concentrations in the NFCO-HF group were higher than the concentrations in all other groups (*p* < 0.05), suggesting that PWHF led to hyperinsulinemia. Specifically, insulin concentrations in this group was 2.3 times higher than concentrations in the NFCO-NF (absolute control) animals (*p* < 0.01), while concentrations in the other PWHF groups did not differ than the NFCO-NF group (Table 1). Leptin concentrations were elevated in both the HFCO-HF and NFCO-HF groups compared to the NFCO-NF group (*p* < 0.05), demonstrating the hyperleptinemia due to PWHF. Interestingly, the leptin concentrations in the choline supplemented HFCS-HF and NFCS-HF groups did not differ than the NFCO-NF group. Moreover, the HFCS-HF group tended to have lower leptin levels than the NFCO-HF group (*p* = 0.05), suggesting that prenatal choline supplementation alleviated the effect of PWHF on increasing serum leptin levels. Other serum markers, such as adiponectin, triglycerides, or FFAs did not differ among the groups.

Female offspring had similar concentrations of these markers among all groups, except that the NFCO-HF group also had higher concentrations of leptin than the NFCO-NF group (*p* = 0.04).

### 3.6. Genes Involved with Insulin Signaling Are Upregulated by HFCS in Visceral Adipose Tissue of Male Offspring

Since leptin was produced by the adipose tissue, and that white adipose tissue (WAT), especially visceral WAT, plays an important role in the development of insulin resistance and glucose intolerance, we measured the weight and gene expression in adipose tissues at different locations of the body to evaluate whether the interaction of HF feeding and MCS on male offspring metabolism was mediated through metabolic changes in the adipose. There were no differences in inguinal, visceral (gonadal), or mesenteric fat weight among maternal dietary treatment groups. PWHF feeding increased fat content in all aforementioned locations (*p* < 0.01) compared to PWNF feeding in both male and female offspring (Table S2). Adipose tissue weight to body weight ratios (as a proxy measurement of body composition) were also higher (*p* < 0.001) in the PWHF offspring for visceral and inguinal fat, but not for mesenteric fat.

We further investigated differential gene expression in the visceral (gonadal) adipose tissue in these offspring, including the expression of *Lep* (leptin), *Pparg* (peroxisome proliferator activator receptor gamma) and *Irs1* (insulin receptor substrate 1) which enhance insulin signaling in WAT, *Retn* (resistin) which enhances liver glucose output and is elevated during obesity, *Rbp4* (retinol binding protein 4) which inhibits liver insulin action, and *Glut4* (glucose transporter 4) which is the transporter that mediates blood glucose uptake into adipocytes in response to insulin.

**Figure 5:**
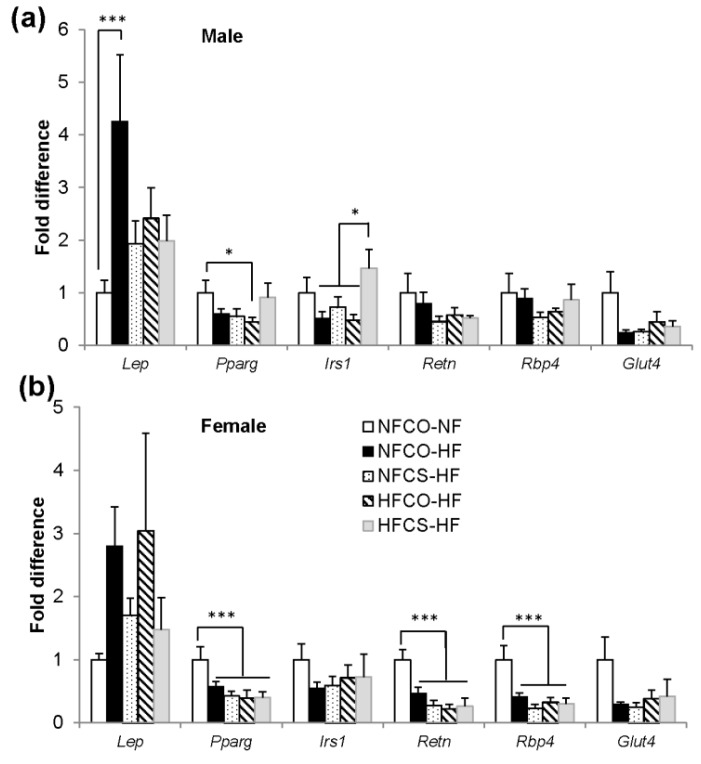
Visceral adipose tissue mRNA expression of mouse offspring after the 6-week post-weaning feeding in (**a**) male and (**b**) female offspring. *n* = 6 for the NFCO-NF group and *n* = 7 for the postnatal HF groups for each sex. Data were analyzed using the general linear model. Values represent means ± SEM. *, *p* < 0.05; ***, *p* < 0.001; CO, untreated control without choline; CS, choline supplemented; *Glut4*, glucose transporter 4; HF, high fat; *Irs1*, insulin receptor substrate 1; NF, normal fat; *Pparg*, peroxisome proliferator-activated receptor gamma; *Rbp4*, retinol binding protein 4; *Retn*, resistin.

In male offspring, we found that the NFCO-HF group had higher *Lep* expression than the NFCO-NF group (*p* = 0.004) (Figure 5a), consistent with the higher leptin in circulation described previously. Prenatal choline supplementation in HF dams (HFCS-HF) led to higher expression of *Irs1* than the other PWHF groups (*p* = 0.024) (Figure 5a). While the HFCO-HF group had lower expression of *Pparg* than NFCO-NF (*p* = 0.039), MCS in the HFCS-HF group tended to eliminate this decrease in gene expression (*p* = 0.06, HFCS-HF versus HFCO-HF) (Figure 5a). Overall, these gene expression patterns were consistent with enhanced insulin signaling by HFCS. All PWHF groups tended to have lower expression of *Glut4* than the NFCO-NF group (*p* = 0.07), while there were no differences in *Retn* or *Rbp4* expression among the groups (Figure 5a).

In female offspring, PWHF led to lower expression of *Pparg* (*p* = 0.007), *Retn* (*p* < 0.001), and *Rbp4* (*p* = 0.001) than the NFCO-NF group, while neither maternal HF nor choline supplementation led to differences in responses to PWHF feeding (Figure 5b).

Lastly, since inflammation impairs insulin signaling, we also examined the expression of *Tnfa* and *Mcp1* in WAT. However, these genes were not differentially expressed among the groups in either male or female offspring (Figure S2).

## 4. Discussion

Maternal HF feeding has been demonstrated to alter various aspects of offspring metabolism, such as adiposity, glucose tolerance, insulin resistance, and hepatic steatosis [7,8,9,11]. Our study demonstrated an interesting interaction of maternal choline supplementation with HF feeding during gestation, where HFCS leading to improved indices in glucose homeostasis in male offspring when they were subjected to HF feeding in postnatal life. The decrease in leptin secretion and upregulation of insulin signaling related gene in the visceral adipose tissue of HFCS male offspring provide a possible mechanism of action. There were notable sexually dimorphic responses to maternal and postnatal dietary treatments.

In this study, male offspring exposed to both maternal HF feeding and choline supplementation (HFCS) demonstrated lower fasting blood glucose and better glucose tolerance after 6-week post-weaning HF feeding compared to control (NFCO) offspring that were exposed to a normal fat and non-choline supplemented environment during gestation. However, neither HF feeding nor choline supplementation during the prenatal period alone modified blood glucose control in the postnatal life. This observation was consistent with our previous studies of fetuses exposed to the same maternal feeding model, where HFCS dams prevented fetal overgrowth in mid-gestation [4] and reduced fetal adiposity in late-gestation [5], while HF alone led to fetal overgrowth and excess adiposity, and that choline supplementation in the normal fat (control) environment had no impact on growth or adiposity. Our findings demonstrate for the first time that the metabolism-modifying effect of choline supplementation under the maternal HF condition seems to be maintained throughout the prenatal and postnatal period in male offspring.

The interaction between HF-induced obesity or metabolic perturbations and choline metabolism is reported in several other studies. HF feeding during murine gestation led to greater food intake and weight gain, and DNA hypomethylation in the prefrontal cortex of offspring, whereas methyl donor supplementation [including choline] attenuated these alterations [31]. Methyl donor supplementation during the perinatal period reduced leptin secretion in rat offspring [32], while supplementing methyl donors to HF-fed or high-fat-sucrose-fed dams protected offspring from increased weight gain [33], adiposity [33], and steatosis [34]. In addition to its role as a methyl donor, choline is also a lipotropic factor that facilitates lipid export from the liver by serving as an essential component on the VLDL membrane. The obesogenic diet-induced steatosis in rodents is relieved by choline or multiple methyl donor supplementation [35,36] which in turn supports the elevated demand for choline under an obesogenic environment. In humans, a few studies have associated obesity and diabetes with altered choline metabolite status, such as lower circulating choline and betaine [the oxidized metabolite of choline] concentrations with higher body mass index [BMI] and total body fat in men [37], and lower betaine concentrations in women with GDM [38]. Higher choline and betaine intake were also correlated with better body composition and lower insulin resistance in the population of Newfoundland, Canada [39,40].

The improvement of blood glucose control in male offspring by maternal HFCS seems to be independent of modifications in liver metabolism. We did not observe signs of triglyceride overaccumulation in the liver from any maternal or postnatal feeding groups in male offspring. The postnatal HF groups had lower expression of lipogenic genes (*Acc2*, *Scd1*) possibly to reduce lipid synthesis, thereby compensating for the higher lipid influx and avoiding triglyceride accumulation in the liver. These results suggest that our current model of 6 weeks of HF feeding may not be enough to induce significant dysregulated lipid metabolism in the liver of male offspring. Nevertheless, significant glucose intolerance was seen at this time point in all postnatal HF groups and was moderately dampened by prenatal choline supplementation of HF dams (HFCS), suggesting that alterations in extrahepatic tissue likely contributed to the disturbance in glucose homeostasis. The dissociation between hepatic phenotypes and blood glucose control in mice was noted by others [41]. A previous study suggests [7] that WAT is the most sensitive to postnatal HF feeding in their model of maternal obesity. In that study, markers of insulin signaling were altered in WAT while static measurements of liver insulin signaling and gluconeogenesis were not modified by the postnatal HF diet [7]. Indeed, we observed that serum leptin levels were increased by post-weaning HF feeding, while maternal choline supplementation in HF dams mitigated the surge in leptin levels in the male offspring. Elevated leptin during obesity is a known risk factor and contributor to insulin resistance and glucose intolerance by suppressing proinsulin synthesis and insulin secretion [42]. Our results suggest that the effect of maternal choline supplementation on glucose homeostasis may be mediated in part through its action on the adipo-insular axis of the offspring. Gene expression analysis in gonadal fat identified higher expression of *Irs1* in the HFCS-HF male offspring than other post-weaning HF groups. IRS1 is a substrate of the insulin receptor tyrosine kinase and has a critical role in insulin signaling [43]. *IRS1* transcription is downregulated in adipose tissue of both rodents and human patients with type 2 diabetes [43,44,45]. Increasing *Irs1* transcription in adipose by HFCS may enhance peripheral insulin signaling and thus improve glucose tolerance. Further analysis on the protein phosphorylation activation of insulin signaling would help confirm this mechanism. Pro-inflammatory cytokines produced by macrophages, such as TNF-α, are reported to impair insulin signaling by increasing IRS-1 serine phosphorylation [46]. Although we did not observe differences in pro-inflammatory cytokine expression in VAT, assays that specifically examine macrophage infiltration and cytokine production can further delineate whether inflammation contributes to the glucose intolerance in HF fed male offspring. In addition to the adipose tissue, the skeletal muscle also plays a critical role in peripheral insulin sensitivity, by serving as a major source of glucose reservation. Whether insulin signaling and glucose uptake in the skeletal muscle are modified by HF feeding and choline supplementation warrants further investigation [47].

Although an array of studies demonstrated that maternal HF-feeding results in higher weight gain and adiposity in rodent offspring [48,49,50,51,52], surprisingly, our study indicated that HFCO offspring had lower weight at weaning. Although post-weaning HF feeding allowed these offspring to gradually catch up in weight, the post-weaning NF-fed male pups exposed to maternal HF feeding prenatally (HFCO-NF) remained lower in weight than the other groups. We also did not observe any added adverse metabolic disturbance from HFCO compared to the control NFCO offspring. The discrepancy between our findings and previous study results may be due to the 60% kcal fat diet that we used in the current study compared to the 45% kcal HF diet and Western-style Diet with added cholesterol used in other maternal HF feeding studies [48,49,50,51,53]. The 60% kcal HF diet has more fat but less carbohydrate (20% versus 30–50% kcal in the form of sucrose or fructose) than those diets [54,55]. As mentioned in Materials and Methods, we chose the 60% kcal fat diet since it can stably induce the obesity and GDM phenotypes in dams and offspring. In our previous studies using the same 60% kcal HF diet to examine its influence on fetal growth, we found higher fetal weight in mid-gestation and greater fetal whole-body adiposity in late gestation [4,5]. These observations suggest that the faltering in the growth of HFCO offspring likely occurred during lactation. The HF diet was not likely to cause low protein and micronutrient consumption in dams since the NF and HF diets have the same amount of protein and micronutrients at the same calorie level. However, the HF, obesogenic diet may have altered milk composition of the dams and affected proper nutrition for the pups. A study using a similar 60% kcal HF diet during lactation demonstrated that HF dams had lower saturated fat and higher unsaturated fat contents in their milk [56]. Pups nursed by HF dams were also protected from obesity when they were challenged with a HF diet post-weaning [56]. In summary, these results highlight the importance of careful characterization of maternal HF-feeding models and their metabolic impacts on offspring at different time points. Interestingly, choline supplementation attenuated the faltered growth in offspring due to maternal HF feeding (HFCS versus HFCO), which again underscores the interaction between choline supplementation and HF and that choline may mitigate the influence of maternal HF on offspring metabolism.

While choline supplementation and HF feeding early in life interacted to influence glucose homeostasis in male offspring, such an interaction was not observed in female offspring. Female offspring were also relatively more protected from high blood glucose and altered serum hormones triggered by post-weaning HF feeding, though the post-weaning HF groups (excluding HFCO-HF) had higher liver triglyceride contents than the absolute control (NFCO-NF). The sexually dimorphic response to maternal exposure and greater resistance to metabolic disturbance due to maternal obesity in females are well-documented in literature [2,8,31,49,57,58], which is possibly related to differential hormonal production, metabolic regulation, and gut microbiome ecology in the two sexes. For instance, sexual hormones play an indirect role to the dysregulation of adipokines during the development of obesity and diabetes, possibly resulting in a stronger correlation between elevated leptin levels and diabetes risk in males versus females [59].

## 5. Conclusions

In conclusion, this study discovered that maternal choline supplementation under the condition of HF feeding can alleviate blood glucose disturbance in male offspring later in life. The influence of early choline exposure may be partly mediated through the improvement in adipose tissue metabolism.

## Figures and Tables

**Table 1:** Metabolic markers after 6-week post-weaning feeding ^1^.

		NFCO-NF	NFCO-HF	NFCS-HF	HFCO-HF	HFCS-HF	*p* Value
	Male offspring						
Serum	Insulin (ng/mL)	0.66 ± 0.21 ^a^	2.15 ± 0.40 ^b^	1.37 ± 0.58 ^a^	1.02 ± 0.16 ^a^	1.43 ± 0.18 ^a^	0.003
	Leptin (ng/mL)	1.83 ± 0.41 ^a^	16.64 ± 4.55 ^b^	10.23 ± 3.36 ^a,b^	13.21 ± 3.27 ^b^	8.21 ± 1.81 ^a^	0.045
	Adiponectin (μg/mL)	55.0 ± 17.3	133.0 ± 91.9	60.7 ± 15.8	67.2 ± 23.1	121.4 ± 76.6	0.796
	Triglycerides (mg/dL)	76.9 ± 10.8	78.8 ± 9.4	89.9 ± 9.4	86.5 ± 9.4	81.7 ± 9.4	0.872
	Free fatty acids (mmol/L)	0.80 ± 0.09	0.72 ± 0.08	0.88 ± 0.08	0.81 ± 0.07	0.76 ± 0.07	0.631
Liver	Triglycerides (mg/g tissue)	6.07 ± 1.90	10.55 ± 1.64	8.99 ± 1.64	8.14 ± 1.64	9.04 ± 1.64	0.507
	Female offspring						
Serum	Insulin (ng/mL)	0.52 ± 0.03	0.60 ± 0.03	0.53 ± 0.03	0.59 ± 0.08	0.62 ± 0.10	0.750
	Leptin (ng/mL)	2.14 ± 0.60 ^a^	7.74 ± 1.88 ^b^	5.79 ± 0.30 ^a^	4.39 ± 1.16 ^a^	4.24 ± 1.21 ^a^	0.069
	Adiponectin (μg/mL)	31.1 ± 11.3	45.0 ± 11.5	28.8 ± 6.8	47.9 ± 15.9	86.0 ± 46.3	0.500
	Triglycerides (mg/dL)	101.2 ± 15.1	79.2 ± 13.1	82.2 ± 14.0	83.2 ± 12.3	68.5 ± 13.1	0.609
	Free fatty acids (mmol/L)	1.48 ± 0.23	0.94 ± 0.23	1.10 ± 0.19	1.07 ± 0.19	0.73 ± 0.19	0.204
Liver	Triglycerides (mg/g tissue)	6.36 ± 1.43 ^a^	10.66 ± 1.23 ^b^	11.03 ± 1.32 ^b^	6.81± 1.16 ^a^	10.72 ± 1.23 ^b^	0.023

^1^ Different diets were fed to dams 4 weeks before timed-mating and throughout gestation. *n* = 6 in the NFCO-NF group for each sex; *n* = 8 in the other groups for each sex. Data were analyzed using the general linear model. ^a,b^ different letters represent statistical significance between groups in post-hoc analyses. Values represent means ± SEM. HF, high-fat; NF, normal-fat; CO, untreated control without choline; CS, choline supplemented.

## Supplementary Materials

The following are available online at https://www.mdpi.com/2072-6643/12/1/144/s1, Figure S1: Liver mRNA expression of mouse offspring after the 6-week post-weaning feeding in male and female offspring., Figure S2: Visceral white adipose tissue mRNA expression of mouse offspring after the 6-week post-weaning feeding in male and female offspring., Table S1: Primers used for real-time PCR., Table S2: Tissue fat content in mouse offspring exposed to different post-weaning diets for 6 weeks.
