# Supplementary material for: Prenatal Choline Supplementation during High-Fat Feeding Improves Long-Term Blood Glucose Control in Male Mouse Offspring
Source: Nutrients. 2020 Jan 4;12(1):144. doi: 10.3390/nu12010144 (PMC7019888; doi:10.3390/nu12010144)
Supplement: Supplementary file 1 [file nutrients-12-00144-s001.zip › Figure S1.docx]

**
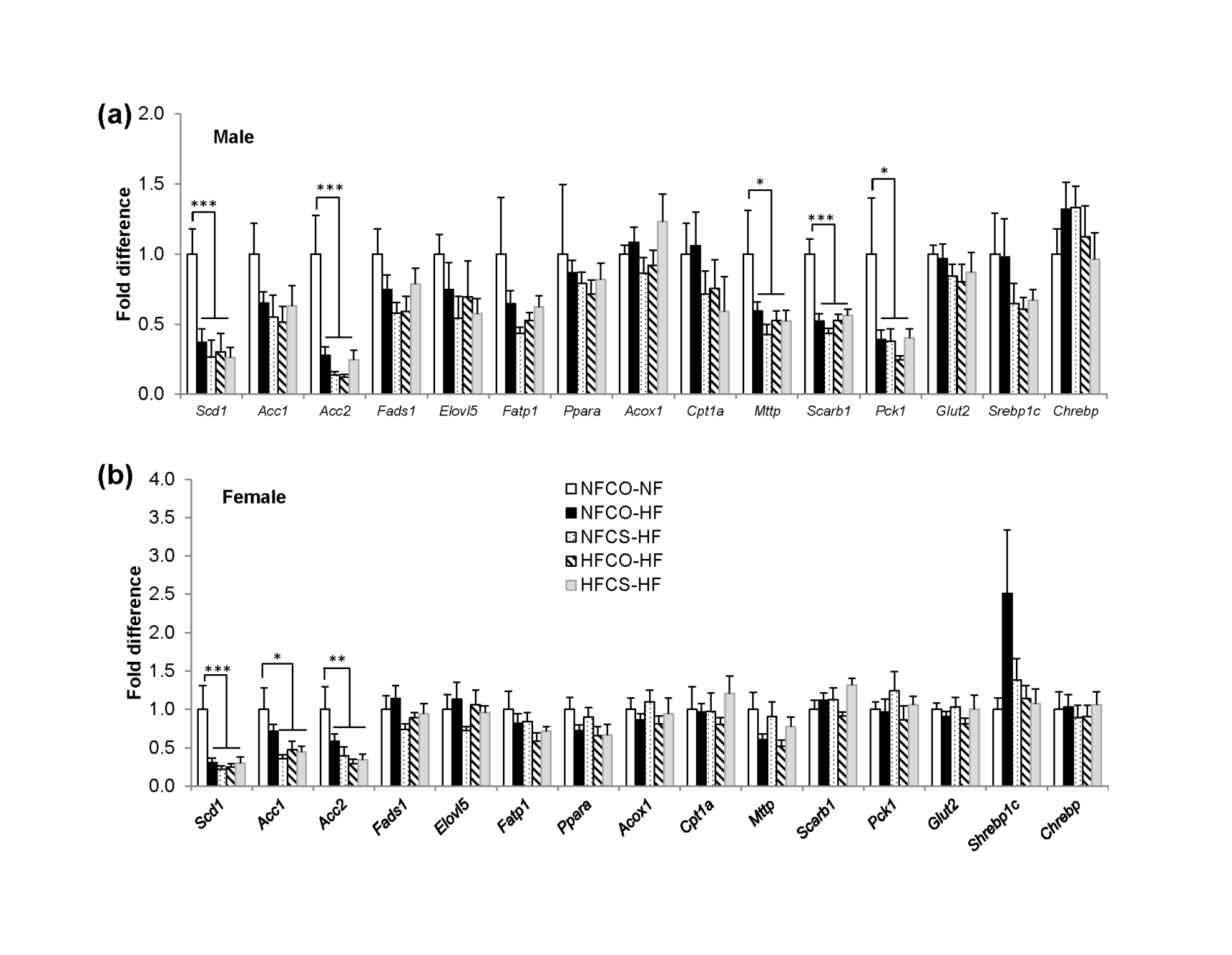
Figure S1.** Liver mRNA expression of mouse offspring after the 6-week post-weaning feeding in (**a**) male and (**b**) female offspring. n=6 for the NFCO-NF group and n=7-9 for the postnatal HF groups for each sex. Data were analyzed using the general linear model. Values represent means ± SEM. *, P < 0.05; **, P < 0.01; ***, P < 0.001; *Acc*, acetyl-CoA carboxylase; *Acox1*, peroxisomal acyl-coenzyme A oxidase 1; *Chrebp*, carbohydrate-response element-binding protein; CO, untreated control without choline; *Cpt1a*, carnitine palmitoyltransferase 1a; CS, choline supplemented; *Elovl5*, ELOVL fatty acid elongase 5; *Fads1*, fatty acid desaturase 1; *Fatp1*, fatty acid transporter 1; *Glut2*, glucose transporter 2; HF, high fat; *Mttp*, microsomal triglyceride transfer protein; NF, normal fat; *Pck1*, phosphoenolpyruvate carboxykinase 1; *Ppara*, peroxisome proliferator-activated receptor α; *Scarb1,* scavenger receptor class B member 1; *Scd1*, stearoyl-Coenzyme A desaturase 1; *Srebp1c*, sterol regulatory element-binding transcription factor 1c.
