# Supplementary material for: Prenatal Choline Supplementation during High-Fat Feeding Improves Long-Term Blood Glucose Control in Male Mouse Offspring
Source: Nutrients. 2020 Jan 4;12(1):144. doi: 10.3390/nu12010144 (PMC7019888; doi:10.3390/nu12010144)
Supplement: Supplementary file 1 [file nutrients-12-00144-s001.zip › Figure S2.docx]

**
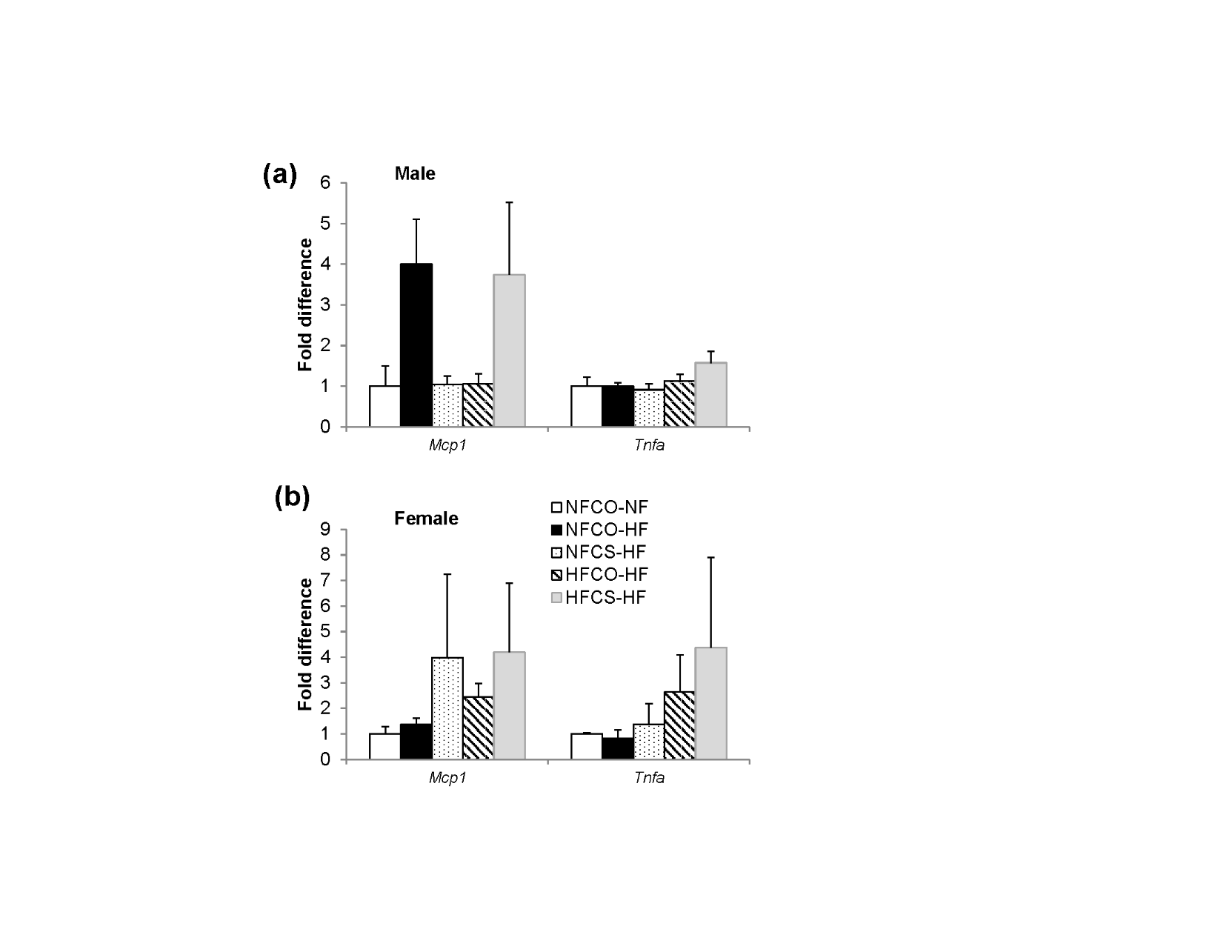
**

**Figure S2.** Visceral white adipose tissue mRNA expression of mouse offspring after the 6-week post-weaning feeding in (**a**) male and (**b**) female offspring. n=6 for the NFCO-NF group and n= 7 for the postnatal HF groups for each sex. Data were analyzed using the general linear model. Values represent means ± SEM. CO, untreated control without choline; CS, choline supplemented; HF, high fat; *Mcp1*, monocyte chemoattractant protein-1; NF, normal fat; *Tnfa*, tumor necrosis factor alpha.
