# Supplementary material for: Prenatal Choline Supplementation during High-Fat Feeding Improves Long-Term Blood Glucose Control in Male Mouse Offspring
Source: Nutrients. 2020 Jan 4;12(1):144. doi: 10.3390/nu12010144 (PMC7019888; doi:10.3390/nu12010144)
Supplement: Supplementary file 1 [file nutrients-12-00144-s001.zip › Table S1.docx]

**Table S1.** Primers used for real-time PCR^1^

| Gene Symbol | Forward Primer | Reverse Primer |
| --- | --- | --- |
| *Acc1* | 5’ GGAGCAGAGAACCTTCGGGG 3’ | 5’ CGGACAAGGTAAGCCCCAAT 3’ |
| *Acc2* | 5’ AGGCAGTCCGCAGGGTGATG 3’ | 5’ CCGCCTTACGCTGCAGGGTG 3’ |
| *Acox1* | 5’ GAAAGCCTAACCGAAGCATA 3’ | 5’ CCTCCTTGCTTTTTCTGTGA 3’ |
| *Actb* | 5’ TAAGGCCAACCGTGAAAAGA 3’ | 5’ TGCCTGTGGTACGACCAGAG 3’ |
| *Chrebp* | 5’ AAGTCCACCCACCTTGGCCT 3’ | 5’ AGGCTCCAGTGCTTGCTCAG 3’ |
| *Cpt1a* | 5’ GCTCTACATCACCCCAACCCAT 3’ | 5’ GAGGGGAATTGTAGATCCCAGA 3’ |
| *Elovl5* | 5’ GGGTGGCTGTTCTTCCAGATTG 3’ | 5’ GTGGCCCTTCAGGTGGTCTTTC 3’ |
| *Fads1* | 5’ GCCTTCAACAACTGGTTCAGTG 3’ | 5’ CGTACTTGGCGCACAGGGATTG 3’ |
| *Fatp1* | 5’ GGCCACCATTCCTACAGCAT 3’ | 5’ CCACCGTCAACCCGTAGATG 3’ |
| *Glut2* | 5’ GCCAATTACCGACAGCCCATCC 3’ | 5’ GATGCCAGCTGTCTGAAAAATGC 3’ |
| *Glut4* | 5’ TTGCTCCAGCTCCTGGGCAG 3’ | 5’ CCCCAGCCGACTCGAAGATG 3’ |
| *Irs1* | 5’ AGACGCTCCAGTGAGGATTTAA 3’ | 5’ GGATTTGCTGAGGTCATTTAGG 3’ |
| *Lep* | 5’ CCAGAAAGTCCAGGATGACACC 3’ | 5’ CCCAGGAATGAAGTCCAAGCCA 3’ |
| *Mcp1* | 5’ CACCAGCAAGATGATCCCAATG 3’ | 5’ GACCTCTCTCTTGAGCTTGGTG 3’ |
| *Mttp* | 5’ GCCACCACTGTTCTCCAGAGAT 3’ | 5’ TTGTGCGCACCGTCTTCTCATG 3’ |
| *Pck1* | 5’ GAGGAGGATTTTGAGAAAGCG 3’ | 5’ AGCTCGATGCCGATCTTTG 3’ |
| *Ppara* | 5’ AATGCCTTAGAACTGGATACA 3’ | 5’ GAGCTTAAGCACGTGCACAA 3’ |
| *Pparg* | 5’ TGCCTTGCTGTGGGGATGTC 3’ | 5’ AGACTCTGGGTTCAGCTGGT 3’ |
| *Rbp4* | 5’ CGCTGAGTTTTCTGTGGACGAG 3’ | 5’ CTTGGCAGGATCTTCAGTGTCT 3’ |
| *Retn* | 5’ TGGCTTAAATTGCTGGACAGTC 3’ | 5’ TTCACGAATGTCCCACGAGC 3’ |
| *Scarb1* | 5’ TCACTGGGATCCCCATGAAC 3’ | 5’ CACAGCAACGGCAGAACTAC 3’ |
| *Scd1* | 5’ CGCCCCTACGACAAGAACATTC 3’ | 5’ TGGCAGAGTAGTCGAAGGGG 3’ |
| *Srebp1c* | 5’ CATGGACGAGCTGGCCTTC 3’ | 5’ ACTGTCTTGGTTGTTGATGAGC 3’ |
| *Tnfa* | 5' AGAAGTTCCCAAATGGCCTCCC 3' | 5’ CGTTGGCCAGGAGGGCGTT 3’ |

^1^Abbreviations: *Acc1*, acetyl-CoA carboxylase 1; *Acox1*, peroxisomal acyl-coenzyme A oxidase 1; *Actb*, beta-actin; *Chrebp*, carbohydrate-response element-binding protein; Cpt1a, carnitine palmitoyltransferase 1a; *Elovl5*, ELOVL fatty acid elongase 5; *Fads1*, fatty acid desaturase 1; *Fatp1*, fatty acid transporter 1; *Glut2*, glucose transporter 2; *Glut4*, glucose transporter 4; *Irs1*, insulin receptor substrate 1; *Lep*, leptin; *Mcp1*, monocyte chemoattractant protein-1; *Mttp*, microsomal triglyceride transfer protein; *Pck1*, phosphoenolpyruvate carboxykinase 1; *Ppara*, peroxisome proliferator-activated receptor α; *Pparg*, peroxisome proliferator-activated receptor gamma; *Rbp4*, retinol binding protein 4; *Retn*, resistin; *Scarb1*, scavenger receptor class B member 1; *Scd1*, stearoyl-Coenzyme A desaturase 1; *Srebp1c*, sterol regulatory element-binding transcription factor 1c; *Tnfa*, tumor necrosis factor alpha.
