# Supplementary material for: Prenatal Choline Supplementation during High-Fat Feeding Improves Long-Term Blood Glucose Control in Male Mouse Offspring
Source: Nutrients. 2020 Jan 4;12(1):144. doi: 10.3390/nu12010144 (PMC7019888; doi:10.3390/nu12010144)
Supplement: Supplementary file 1 [file nutrients-12-00144-s001.zip › Table S2.docx]

**Table S2.** Tissue fat content in mouse offspring exposed to different post-weaning diets for 6 weeks^1^

|  | **Male** | |  | **Female** | |
| --- | --- | --- | --- | --- | --- |
| **Post-weaning Diet** | **NF** | **HF** |  | **NF** | **HF** |
| Gonadal Fat (g) | 0.36 ± 0.06 | 1.06 ± 0.05** |  | 0.18 ± 0.04 | 0.48 ± 0.03** |
| Inguinal Fat (g) | 0.18 ± 0.02 | 0.37 ± 0.01** |  | 0.15 ± 0.01 | 0.28 ± 0.01** |
| Mesenteric Fat (g) | 0.12 ± 0.02 | 0.17 ± 0.01** |  | 0.08 ± 0.01 | 0.13 ± 0.01** |
| Gonadal fat/body weight | 0.015 ± 0.0008 | 0.035 ± 0.002** |  | 0.010 ± 0.0006 | 0.021 ± 0.001** |
| Inguinal fat/body weight | 0.008 ± 0.0003 | 0.012 ± 0.0005** |  | 0.009 ± 0.0005 | 0.013 ± 0.0004** |
| Mesenteric fat/body weight | 0.005 ± 0.0007 | 0.006 ± 0.0003 |  | 0.005 ± 0.0003 | 0.006 ± 0.0005 |

^1^ Prenatal diet did not affect the fat content in these locations. Therefore, data were averaged among all prenatal diet groups. Data were analyzed using the general linear model. n = 48 in the NF group of each sex; n = 75-77 in the HF group of each sex. ****** HF versus NF, *P* < 0.01; Values represent means ± SEM. HF, high-fat; NF, normal-fat
